# First case report on the occurrence of *Trypanosoma evansi* in a Siam B Mare in Kelantan, Malaysia

**DOI:** 10.1002/vms3.379

**Published:** 2020-11-07

**Authors:** Nur Zul Izzati Mohd Rajdi, Mimi Armiladiana Mohamad, Li Peng Tan, Siew Shean Choong, Mohd Farhan Hanif Reduan, Ruhil Hayati Hamdan, C. W. Salma C. W. Zalati

**Affiliations:** ^1^ Clinical Department Faculty of Veterinary Medicine Universiti Malaysia Kelantan Pengkalan Chepa Kelantan Malaysia; ^2^ Paraclinical Department Faculty of Veterinary Medicine Universiti Malaysia Kelantan Pengkalan Chepa Kelantan Malaysia

**Keywords:** diagnosis, equine trypanosomiasis, histopathology, molecular confirmation, Surra

## Abstract

This is the first case report for the positive *Trypanosoma evansi* incident in Kelantan, Malaysia confirmed through protozoa detection in a Siam B mare. The horse was presented with complaints of lethargy and inappetence and it collapsed on the day of visit. Normal saline and dextrose solution were administered intravenously, while multivitamins and nerve supplements were given intramuscularly to stabilise the horse before further treatment. Haematological findings showed normocytic hypochromic anaemia and are suggestive of regenerative anaemia. Thin blood smear and examination revealed the presence of *Trypanosoma* sp., and it was confirmed as *T. evansi* through molecular identification. The horse was found dead 2 days after and post‐mortem was conducted. Histopathology revealed that the horse had developed a neurological form of the disease, besides the detection of the protozoa in heart, spleen and kidney tissue. This first positive Surra case in Kelantan, Malaysia, that is bordering Thailand confirms the increasing concern of transboundary infections. In conclusion, Surra is a potential emerging disease and should be considered as differential diagnosis in horses with pale mucous membrane. This condition is particularly imperative in horses found in these regions as Surra is endemic.

## Graphical abstract

This first positive Surra case in Siam B mare in Kelantan, Malaysia, that is bordering Thailand confirms the increasing concern of transboundary infections. Thin blood smear and examination revealed the presence of Trypanosoma sp., and it was confirmed as *T. evansi* through molecular identification. Histopathology revealed that the horse developed a neurological form of the disease, besides the detection of the protozoa in heart, spleen and kidney tissue.

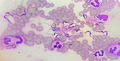

## 1 INTRODUCTION

Pathogenic equine trypanosomiasis is attributed to five haemoflagellate protozoa species of the genus *Trypanosoma* that occur in blood and tissue fluids of infected horses (Luckins, 2010). Specifically, *Trypanosoma evansi* is the causative organism of Surra and has the most extensive host range and geographical distribution worldwide (Büscher et al., 2019; Desquesnes et al., 2013). Surra most often affects horses compared to other animal species, and it may cause rapid fatality in cases of acute and severe infection (World Organisation for Animal Health (OIE), 2013). Mechanical transmission by biting insects is the most important transmission mode of *T. evansi*. The geographical distribution of *T. evansi* is spreading steadily in Asia, possibly due to the abundance of animal reservoir (Desquesnes et al., 2013; Luckins, 1988). In addition, the warm and humid climate in many Asian countries promote the propagation of biting insects involved in mechanical transmissions (Desquesnes et al., 2013). Several outbreaks in horses are recorded every year in Thailand and are frequently fatal (Desquesnes et al., 2013). This condition agrees with the low prevalence of positive animals in serological screening as survivours are low after outbreaks (Kongkaew et al., 2012). In Malaysia, although trypanosomiasis caused by *T. evansi* has been known for years with reported outbreaks in deer, cattle, buffaloes and pigs (Arunasalam et al., 1995; Adrian et al., 2010; Cheah et al., 1999; Lee et al., 1991; Nurulaini et al., 2013), it was given little attention and a national survey has not been organised to evaluate its impact. Previously, merely two publications demonstrated the presence of this parasite in horses (Ikede et al., 1983; Ng & Vanselow, 1978). The most recent study in Peninsular Malaysia reported an overall molecular prevalence and seroprevalence of *T. evansi* in horses are 1.14% and 13.90%, respectively (Elshafie et al., 2013a, 2013b). Kelantan, a state bordering Thailand, has not recorded the presence of *T. evansi*, making this case the first report of *T. evansi* in Kelantan since it was first detected in Malaysia back in 1903 (Fraser & Symond, 1909). The equine industry is expanding in Malaysia and horses are imported or illegally smuggled from countries that are no longer free from *T. evansi*. There are mandatory tests for imported horse in Malaysia, such as Equine Infectious Anaemia (EIA), Equine Herpes Virus (EHV), Japanese Encephalitis, Strangles and Nipah (Department of Veterinary Malaysia, 2011), but not Surra. Consequently, horses entering the country could serve as carriers and introduce these protozoa to a susceptible pool of naive animals. Due to its high mortality in horses and possible zoonotic concern (Powar et al., 2006), greater attention should be given to Surra in Malaysia.

## 2 CASE HISTORY

This case involved a 10‐year‐old Siam B mare experiencing inappetence and progressive weight loss for 2 months. The veterinary clinic of Universiti Malaysia Kelantan was called in for treatment and further diagnosis when it finally collapsed due to weakness. Further history taking revealed that the mare is already in poor body condition despite still having a good appetite at the time of purchase. The mare was given symptomatic and supportive treatment during the first visit, while samples were collected for diagnostic workup. However, the animal was found dead 2 days later, on the morning of the planned second visit. Post‐mortem was conducted to determine the cause of death.

## 3 CLINICAL FINDINGS

**FIGURE 1:**
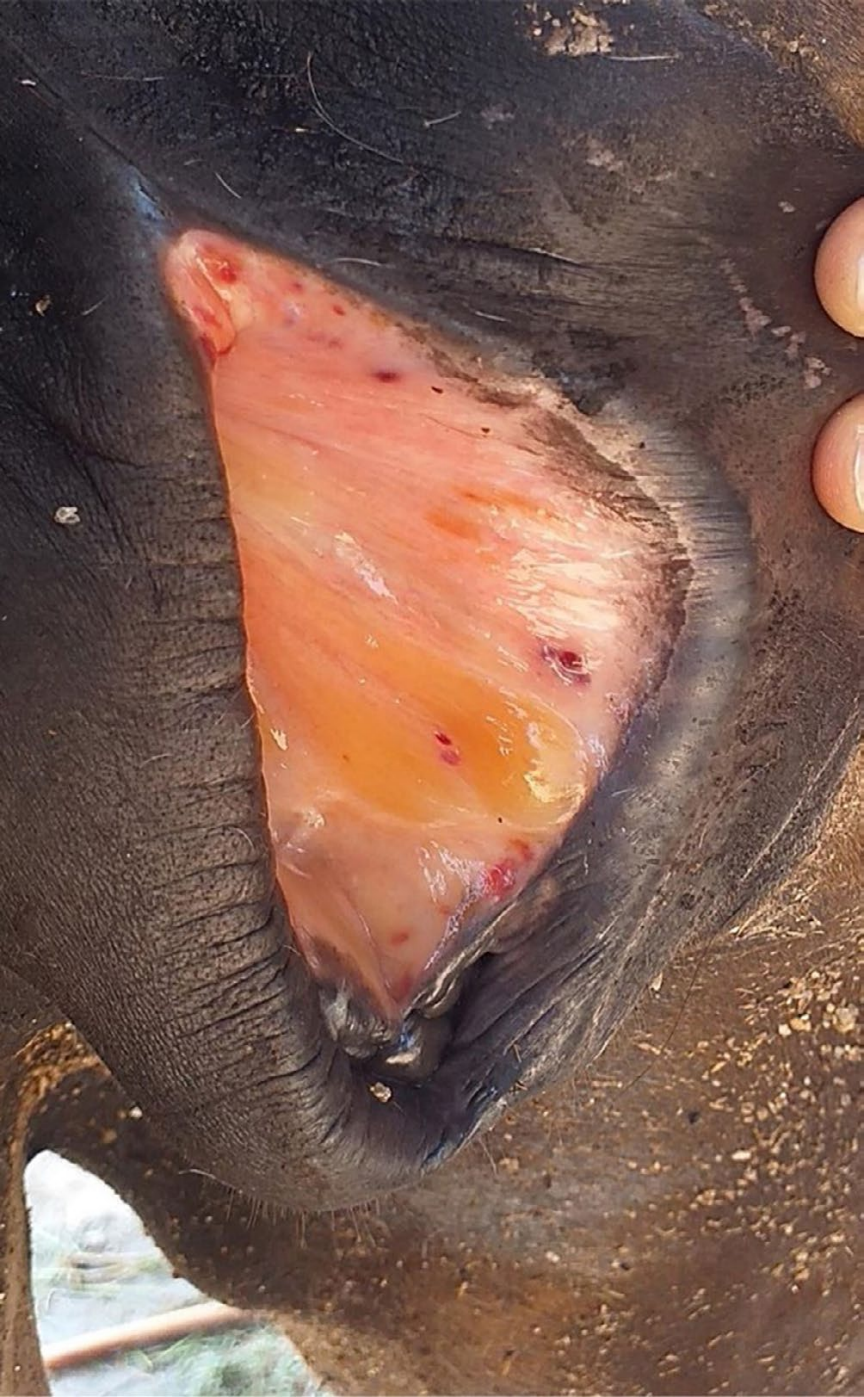
Presence of petechiation and gelatinous material on the vulvar mucosa of the Siam B mare infected with *Trypanosoma evansi*

Upon presentation to the university veterinary clinic, the mare was in lateral recumbency, appeared dull, but was responsive to stimuli. Physical examination revealed a rectal temperature of 39.6°C, respiratory rate of 52 breaths/minute, heart rate of 100 beats/minute and the pulse was strong and regular. The mare was pyrexic with tachypnea and tachycardia. Its body score was 1/5, characterised by a prominent ribcage and pelvic bone indicating emaciation. The animal's dehydration status was 10% with skin tent and capillary refill time (CRT) at 3 s, a delay from the norm and indicated hypovolemic shock. Oral mucous membrane appeared pale pink, while there was the presence of petechiation and gelatinous materials on the vulvar mucosa (Figure 1). Furthermore, urticaria was observed at the right lateral region of the neck, frontal area of the head and on all extremities. Moreover, multiple abrasive wounds were found at the hip joint, shoulder and forehead. These traumatic injuries were most likely sustained when the mare was struggling to stand due to progressive weakness and incoordination. Neurological signs such as staggering gait, dragging of both hindlimbs, hindquarter weakness, incoordination and ataxia were observed in the video captured by the owner before the mare collapsed. Blood was taken from jugular vein in both plain and EDTA tube for thin blood smear examination, complete blood count, serum biochemistry analysis and Polymerase Chain Reaction (PCR). At this point in time, the differential diagnosis for this case was highly suggestive of blood parasite infestation.

## 4 DIAGNOSIS

### 4.1 Haematological and serum biochemical changes

The haematological results demonstrated low red blood cell (RBC) count, mean corpuscular haemoglobin concentration (MCHC), haemoglobin (HGB) level and haematocrit, indicative of normocytic hypochromic anaemia (Table 1). Furthermore, the mare was shown to have leucocytosis and monocytosis as a result of increased activities of the mononuclear phagocyte system to engulf the senescent and dead erythrocytes due to extensive extravascular haemolysis. Conversely, the mean corpuscular volume (MCV) was normal. For serum biochemistry, total bilirubin (TBIL) was slightly elevated, also hypocalcaemia, hypoglycaemia and hypophosphatemia (Table 1).

**TABLE 1:** Hematologic and serum biochemistry results of the Siam B mare infected with *Trypanosoma evansi*

Parameter	Results	Range	Parameter	Results	Range
RBC (10^6^/µl)	**5.1**	6.0–12.0	Total bilirubin (mg/dl)	2.9	0–3.5
HGB (g/dl)	**9.7**	11.0–17.0	Alanine transaminase (µ/l)	15	5–50
HCT (%)	**24.6**	35.0–55.0	γ‐glutamyl transferase (µ/l)	21	0–87
MCV (µm^3^)	**35.7**	34.0–58.0	Creatine kinase (µ/l)	108	10–350
MCH (pg)	24.5	26.0–34.0	Total protein (g/dl)	6.0	5.6–7.9
MCHC (g/dl)	**29.1**	31.0–35.5	Albumin (g/dl)	2.2	1.9–3.2
WBC (10^3^/µl)	**12.7**	4.0–12.00	Globulin (g/dl)	3.8	2.4–4.7
Monocyte (10^3^/µl)	**2.3**	0.1–0.8	Calcium (mg/dl)	**4.3**	10.4–12.9
Lymphocyte (10^3^/µl)	5.1	1.5–5.5	Glucose (µ/l)	**41**	64–150
Granulocyte (10^3^/µl)	5.3	2.0–8.0	Phosphorus (mg/dl)	**1.5**	1.8–5.6
Platelet (10^3^/µl)	191	150–400			

Note

Values out of the normal range for horses are indicated in bold.

### 4.2 Parasite detection

**FIGURE 2:**
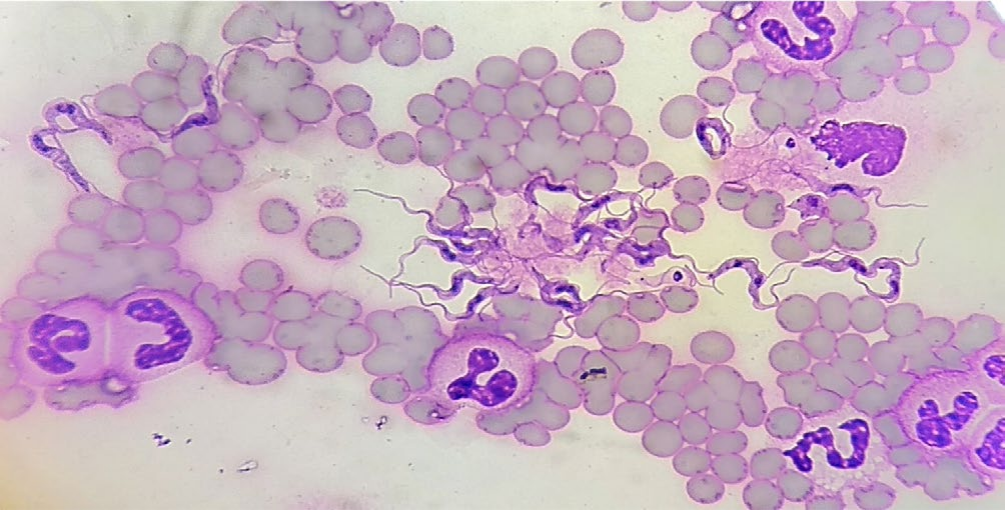
High parasitaemia of *Tryp* *anosoma evansi* trypomastigotes shown in the blood smear. Diff‐quick stain. Scale bar—10 μm

Thin blood smear examination revealed high parasitaemia by *Trypanosoma* spp., characterised by central nucleus, kinetoplast, undulating membrane and flagellum (Figure 2). PCR revealed the mare was negative for both *Theileria equi* and *Babesia caballi* but positive for *Trypanosoma evansi*. Kin1 and Kin2 primers described by Desquesnes et al. (2001) were used. These primers anneal in the conserved regions of the 18S and 5.8S rDNA to amplify the ITS1. Primer sequences are as follows: Kin1, 5′‐GCG TTC AAA GAT TGG GCA AT‐3′ (reverse); Kin2, 5′‐CGC CCG AAA GTT CAC C‐3′ (forward). Thus, the definitive diagnosis for this case is Surra. The sequence information of the protozoa was compared with sequences available in the GenBank of the National Centre for Biotechnological Information (NCBI ‐ www.ncbi.nim.nih.gov) using the Basic Local Alignment Search Tool (BLAST) programme. BLAST analysis revealing 99.59% sequence nucleotide match with *T. evansi* sequences from Bangkok, Thailand (accession no. AY912278.1).

### 4.3 Treatment and outcome

On the day of presentation, the mare was administered with 0.9% sodium chloride (INFUSOL^®^, NS) and 5% dextrose solutions (INFUSOL^®^, DS5) dosed at 20 ml kg^−1^ h^−1^ and 2 ml/kg, respectively, via intravenous route to improve the circulating volume to overcome hypovolemic shock. A total of 20 ml multivitamins and nerve supplement (Biodyl^®^, Merial) was given intramuscularly to stabilise the condition of the horse. Once the confirmative diagnosis of Trypanosomiasis was reached, diminazene aceturate (Barenil^®^) was planned to be administered (7 mg/kg), intramuscularly. However, the mare was found dead by the owner on the day of planned second visit and post‐mortem was performed four hours later after its death.

### 4.4 **Histopathologica**l **findings**

**FIGURE 3:**
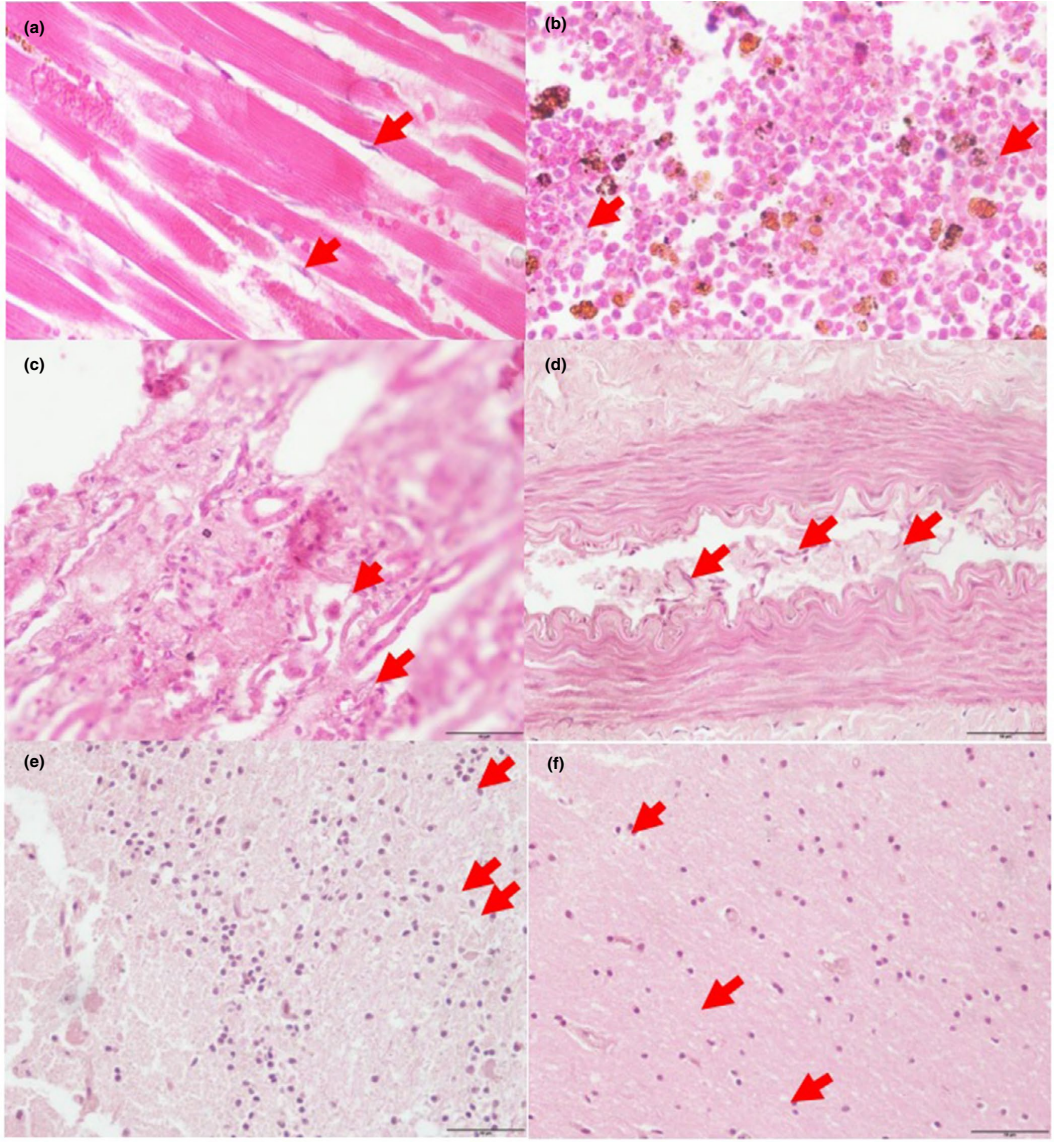
(a) Presence of *Trypanosom* *oa evansi* (arrow) in between the cardiomyocyte and cardiomylysis; (b) Presence of *T. evansi* (arrow) in the spleen; (c) Presence of *T. evansi* (arrow) in the medulla of the kidney; (d) Presence of many trypomastigotes form of *Trypanosoma evansi* (arrow) in the lumen of the blood vessels of the spinal cord; (e) Trypanosomes (arrow) located in the molecular layer of cerebellum; (f) Trypanosomes (arrow) located in the molecular layer of cerebral cortex (H&E staining, 40×) (scale bar: 50 μm)

Histopathological results demonstrated the presence of *T. evansi* between the cardiomyocytes, in the spleen, medulla of the kidney, spinal cord, cerebrum and cerebellum (Figure 3).

## 5 DISCUSSION

The clinical signs observed in this mare concurred with those reported in horse Surra, such as fever, progressive anaemia and lethargy, urticarial plaques on the skin and petechial haemorrhages of the serous membranes staggering gait, dragging of both hind‐limbs, hindquarter weakness, incoordination and ataxia (Marques et al., 2000; World Organisation for Animal Health (OIE), 2013). Also, the infected mare showed similar clinical signs as the experimentally inoculated ponies such as excessive weakness, reduction of appetite and body weight loss (Elshafie et al., 2018).

Haematological findings of the infected mare revealed normocytic and hypochromic anaemia. In contrast, the ponies inoculated with *T. evansi* at day‐16 demonstrated normocytic and normochromic anaemia (Elshafie et al., 2018). Meanwhile, white blood cell parameters in these two cases were leucocytosis and monocytosis. Although the mare had poor body condition persistently for 3 months and became inappetent for 2 weeks after it relocated from Thailand, haematology and clinical signs revealed that its anaemic condition was mild to moderate, which suggested that the mare was in the second phase of anaemia. Typically, this second phase is characterised by low levels parasitaemia, which usually begins between 4 and 6 months after *T. evansi* infection (Jain, 1986). However, high parasitaemia in this case may be due to concentrated blood volume as a result of dehydration. Meanwhile, the first phase of anaemia (acute crises) is generally moderate to severe, with high parasitaemia during the early stage of the disease. The third phase (recovery) will demonstrate low, infrequent or absence of parasitaemia with erythrocyte values returning towards normal (Mbaya et al., 2012).

The progressive anaemia observed in this case is known as anaemia of chronic disease or inflammation is commonly observed in trypanosusceptible animal. It is characterised by enhanced erythrophagocytosis and impaired erythropoiesis due to altered iron haemostasis and persistence release of pro‐inflammatory cytokines such as INF‐γ, TNF, IL‐1 and IL‐6 (Stijlemans et al., 2018). In contrast, some animals may develop trypanotolerant and exhibits anti‐inflammatory responses (Il‐10) and an increase in iron availability for erythropoiesis. This explains the return of erythrocyte values to normal in the chronic stage of the disease.

Moreover, the slight elevation in TBIL may be due to fasting‐induced hyperbilirubinemia in response to the reduced uptake of bilirubin. This reduction is because of competition with the free fatty acids release from tissues to provide energy and to sustain the physiological functions of the body. Hypocalcaemia, hypoglycaemia and hypophosphatemia depicted in the result are higher due to malnutrition. The hypoglycaemic state of the mare also can be attributed to the pathogenesis of the trypanosomes whereby these parasites consume glucose for its own metabolism.

Infiltration and dissemination of *T. evansi* in the central nervous system of the infected animal are often considered as a chronic case by veterinarians. Typical clinical signs of a chronic case include progressive weakness, emaciation, depletion, recurrent fever, enlarged lymph nodes and eventual death of the animal (Berlin et al., 2009; Omer et al., 2007; Saleh et al., 2009; Silva et al., 1995). In the case of acute incidences, the clinical signs are anaemia, high fever, weakness and high mortality in a naïve population (Desquesnes et al., 2013; Silva et al., 1995). Based on the history, clinical signs and histopathological findings for the current case, it was suggestive that the mare was infected with *T. evansi* before it was transported to Kelantan and it was in chronic evolution before death. Additionally, this mare was kept with other horses in Thailand and Malaysia, and high mortality was not reported in both locations. Thus, it is indicative that this case is more probable in a case of chronic trypanosomiasis rather than an acute case.

Comparison has been made with Marques et al. (2000) and Berlin et al. (2009). In general, all the cases were categorised as chronic infection with signs of chronic weight loss, emaciation, poor body condition, pyrexia and showed signs of neurological abnormalities. Neurological signs were more prominent on hind limbs in which weakness, ataxia and incoordination were observed in Marques et al. (2000) and our case, while Berlin et al. (2009) reported partial right‐sided facial paralysis in addition of these clinical signs on all limbs. In contrast, nervous signs were not reported in ponies that received experimental inoculation of *T. evansi* (10^2^ live trypanosomes/kg body weight). Elshafie et al. (2018) believed the absence of clinical signs was due to the short period of infection. The serum biochemistry results, in this case, were inconsistent with Berlin et al. (2009). Hypoproteinemia, hypoalbuminemia and hypercalcemia were reported by Berlin et al. (2009), whereas hypoglycaemia, hypophosphatemia and hypocalcemia were found in this case. Increase of bilirubin or icterus index is often related to trypanosomiasis in horses. An experimental study conducted by Marques et al. (2000) showed an increase in the mean icterus index after inoculation until 7th weeks, then decreased after 9th weeks supported the above statement. Nevertheless, icterus can only be observed in cases of severe haemolysis concurrent with high parasitaemia. Thus, this explained the normal bilirubin level observed in Berlin et al. (2009), while the mare in the current case exhibited the elevated bilirubin level as it was experiencing low parasitaemia stages during the chronic infection period.

Prevention of this disease can be performed by controlling the presence of both agents and vectors. However, the control of the vector is difficult due to the ecology of the vector itself (Howard, 2013; Wilson et al., 2020). But it can be performed by practising strict hygiene of the stable, clearing the surrounding area from bushes and wet areas that can be potential breeding grounds for the vectors and through the use of insecticides (Masrin et al., 2018). Diminazene aceturate is the most widely used curative trypanocide against *T. evansi* infection in horses. The recommended dose of diaminizine aceturate is 7 mg/kg/bodyweight, but this dose might have toxic effects in severely dehydrated animal. Thus, it is recommended to split the dose into two sub boosts of 3.5 mg/kg bodyweight, at 3–5 hr interval. However, in the case of pathogen invasion into the nervous system, this drug is not effective (Desquesnes et al., 2013).

Chronic trypanosomiasis may be under‐reported in Malaysia as animals do not show obvious signs as in acute trypanosomiasis. Although blood smear examination may be relatively easy and cheap to perform, it has a low sensitivity rate as parasitaemia is relatively low in chronic cases (Ramírez‐Iglesias et al., 2011). Definitive diagnosis made through histopathology and PCR was possible because the case was used for veterinary training purposes. In normal circumstances, these diagnostic procedures may not be conducted as an additional cost to the owner is generally not acceptable without strong suspicion of the aetiology. Thus, this may have contributed to the lack of information on *T. evansi* infection in horses in Malaysia. Without updated information on trypanosomiasis, local stud farms and horse owners may be unknowingly threatened by the disease as it is severely pathogenic in horses. Therefore, it is recommended that cases with similar clinical signs should be investigated further and any new additional animals to the herd must be screened.

Molecular characterisation of *T. evansi* is crucial as previous studies have identified multiple strains of the microorganisms isolated from cattle, horses and donkeys with varying virulence from low to high pathogenicity (Mekata et al., 2012; Perrone et al., 2018). Hence, the identification of the species of Trypanosoma is incomplete without the determination of its strain. Such information will be important for treatment and preventive measures for future cases as the severity of the disease directly contributes to the economic impact on the equine industry.

## 6 CONCLUSION

Surra in horses has not been reported in Kelantan priorly, although this state is geographically adjacent to Thailand. This occurrence of *T. evansi* in Kelantan, Malaysia is the first confirmative case of transboundary Surra from Thailand to Malaysia. There are considerable differences in the severity of infection in horses depending on the virulence of the strain and the susceptibility of the host. Substantial loss may ensue if naive populations of horses in Malaysia are infected by *T. evansi* from Thailand, even if Surra is endemic in Malaysia. Thus, trypanosomiasis caused by *T. evansi* remains a potential disease outbreak concern and should be considered in the differential diagnosis whenever pale mucous membrane is observed in horses in this region. The pathogenicity of *T. evansi* infection in horses in the country should be reflected in detail for a better understanding of the host‐parasite relationship. Thereby, this would assist in early diagnosis to reduce economic and resource losses due to this disease.

## ETHICAL ANIMAL RESEARCH

The client signed a release form to consent to post‐mortem and sample collection from his/her animal for diagnostic and learning purposes.

## CONFLICT OF INTEREST

No conflict of interest has been declared.

## AUTHOR CONTRIBUTION

**Nur Zul Izzati Mohd Rajdi:** Investigation; Writing‐original draft. **Mimi Armiladiana Mohamad:** Investigation; Resources. **Siew Shean Choong:** Investigation; Writing‐review & editing. **Mohd Farhan Hanif Reduan:** Formal analysis; Writing‐original draft. **Ruhil Hayati Hamdan:** Formal analysis; Writing‐review & editing. **C.W. Salma C.W. Zalati:** Methodology; Writing‐review & editing. **Li Peng Tan:** Formal analysis; Writing‐original draft.

### PEER REVIEW

The peer review history for this article is available at https://publons.com/publon/10.1002/vms3.379.
